# In vivo assessment of coronary flow and cardiac function after bolus adenosine injection in adenosine receptor knockout mice

**DOI:** 10.14814/phy2.12818

**Published:** 2016-06-14

**Authors:** Bunyen Teng, Stephen L. Tilley, Catherine Ledent, S. Jamal Mustafa

**Affiliations:** ^1^ Department of Physiology & PharmacologyWest Virginia UniversityMorgantownWest Virginia; ^2^ Department of MedicineUniversity of North CarolinaChapel HillNorth Carolina; ^3^ Universite Libre de BruxellesBrusselsBelgium

**Keywords:** Adenosine receptor knockout mice, adenosine receptors, cardiac output, coronary flow, ejection fraction, heart rate, stroke volume, ultrasound

## Abstract

Bolus injections of adenosine and the A_2A_ adenosine receptor (AR) selective agonist (regadenoson) are used clinically as a substitute for a stress test in people who cannot exercise. Using isolated tissue preparations, our lab has shown that coronary flow and cardiac effects of adenosine are mostly regulated by the AR subtypes A_1_, A_2A_, and A_2B_. In this study, we used ultrasound imaging to measure the in vivo effects of adenosine on coronary blood flow (left coronary artery) and cardiac function in anesthetized wild‐type, A_1_ knockout (KO), A_2A_KO, A_2B_KO, A_3_KO, A_1_, and A_3_ double KO (A_1/3_ DKO) and A_2A_ and A_2B_ double KO (A_2A/2B_ DKO) mice in real time. Echocardiographic and Doppler studies were performed using a Visualsonic Vevo 2100 ultrasound system. Coronary blood flow (CBF) baseline data were obtained when animals were anesthetized with 1% isoflourane. Diameter (D) and velocity time integral (VTI) were measured on the left coronary arteries (CBF = ((π/4) × D^2^ × VTI × HR)/1000). CBF changes were the highest within 2 min of injection (about 10 mg/kg). Heart rate, cardiac output, and stroke volume were measured by tracing the left ventricle long axis. Our data support a role for the A_2_ AR in CBF and further support our conclusions of previous studies from isolated tissues. Adenosine‐mediated decreases in cardiac output and stroke volume may be A_2B_ and/or A_3_ AR‐mediated; however, the A_1_ and A_2_ ARs also play roles in overall cardiac function. These data further provide a powerful translational tool in studying the cardiovascular effects of adenosine in disease states.

## Introduction

Intravenous adenosine injection has been used clinically to treat surgical and nerve pain (Sjolund et al. 2001), pulmonary hypertension (Fullerton et al. 1996; Ng et al. 2004), and paroxysmal supraventricular tachycardia (Rankin et al. 1992; Chronister 1993; Lozano et al. 1995). However, the main use of adenosine is for myocardial perfusion imaging in patients who cannot exercise. There are four adenosine receptor (AR) subtypes, A_1_, A_2A_, A_2B_, and A_3_, and all have demonstrated a role in regulating coronary blood flow (CBF) and cardiac function. Studies from our lab and others have demonstrated that the A_2A_ AR is the predominant AR in the coronary circulation, with the A_2B_ AR playing a complimentary role; the A_1_ and A_3_ ARs play only modulating roles in CBF regulation. Regarding their roles in cardiac function, in general, only the A_1_ AR mediates the negative dromotropic and chronotropic properties of adenosine, while the A_1_, A_2A_, and A_2B_ ARs all mediate inotropic properties in some way depending on the species and pathological situation. There are very few reports on the role of the A_3_ AR in cardiac function due to its low expression level.

Because of the availability of many transgenic strains, mice have become one of the most widely used models in medical research. However, their small size makes it very difficult to measure the cardiac function, especially CBF, in live animals. Ultrasound measurement of CBF and cardiac function has been routinely used in humans, and this noninvasive method has also been used in measuring mouse cardiac function in recent years (Katz et al. 2011). Since previous functional studies of ARs have been mostly conducted ex vivo or in vitro using pharmacological tools, the specificity of adenosine analogs to these ARs and their translational relevance have always come into question. Therefore, using genetic AR knockout (KO) mice, we sought to confirm previous findings in isolated tissues using the newly available in vivo ultrasound technique in mice.

## Materials and Methods

### Animals

All the AR KO and DKO mice were obtained from Dr. Stephen Tilley, University of North Carolina Chapel Hill, with the A_2A_ AR KO mice originating from Dr. Catherine Ledent of Universite Libre de Bruxelles, Brussels, Belgium. All KO and DKO mice were of the C57BL/6 (WT) background.

### Assessment of echocardiography and coronary blood flow Doppler measurement

**Figure 1:**
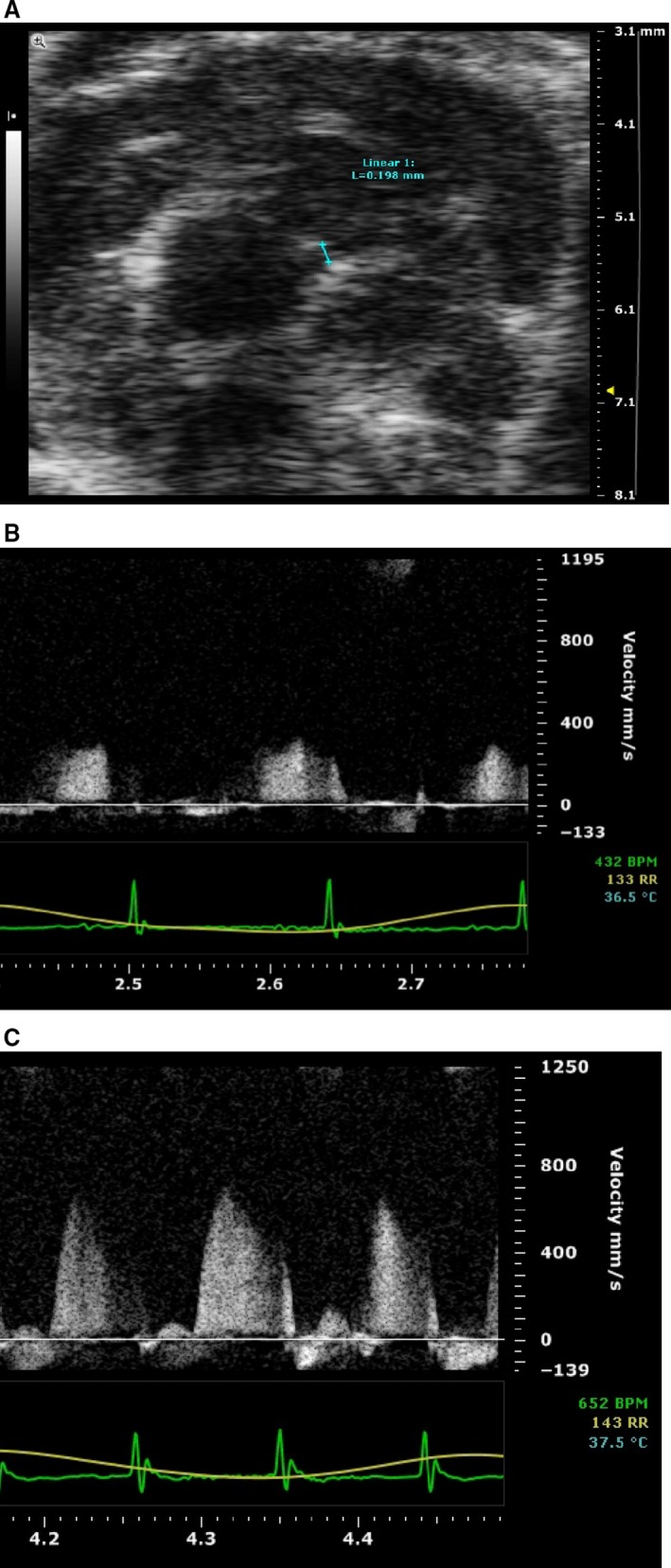
Sample ultrasound images for (A) size of coronary artery; (B) baseline pulse wave Doppler; (C) adenosine‐induced increase in pulse wave Doppler.

Each mouse was anesthetized in an induction chamber with inhalant isofluorane at 3% in 100% oxygen. When fully anesthetized, the mouse was transferred to dorsal recumbency, placed on a heated imaging platform, and maintained at 1–1.25% isofluorane for the duration of the experiment. The hair of the mouse chest wall was carefully removed, and warm electrode gel was applied to the limb leads, allowing for an electrocardiogram and respiration rate to be recorded during ultrasound imaging. A rectal probe was used to monitor body temperature. Ultrasound images were acquired using an MS550D transducer (22–55 MHz) on a Vevo2100 Imaging System (Visual Sonics, Toronto, Ontario, Canada). Placing the transducer to the left of the sternum allowed us to obtain images of the aortic outflow tract, the apex of the heart, and the left ventricle along its longest axis (i.e., long‐axis B‐mode images). Once all long‐axis B‐mode images were attained, the transducer was rotated 90 degrees to acquire short‐axis B‐mode images at the mid‐papillary muscle level, then moved up until the left coronary artery (LCA) was visible for vessel size measurement (Fig. 1A). The transducer was rotated back to the long‐axis parasternal view with the probe lateralized and the ultrasound beam anteriorly tilted. In this image window, the entire LCA, from the aortic sinus to the distal branch site, could be visualized using color Doppler echocardiography. The course of the LCA was typically parallel to the Doppler beam, which also facilitates Doppler measurements without any angle correction. The system was then switched to pulse‐wave Doppler mode with a gate size of 0.65 mm. Coronary flow signals were identified on the Doppler spectral display by flow toward the probe peaking in early diastole, followed by decay, and being minimal during systole as illustrated in Figure 1B. Bolus adenosine injection (0.2 mg/mouse; approximately 10 mg/kg) was made through the femoral vein. The dosage was chosen based upon previous studies (Zhao et al. 2000; Koeppen et al. 2009). The high dose was chosen because we want to make sure that all adenosine receptors get activated and knowing that adenosine has a short half‐life which will reduce its effect over the period of our measurement. The blood flow velocity was measured using Doppler principle to directly measure the flow motion (pulsed‐wave Doppler mode, PW‐mode). It will not pick up tissue motion. Measuring the size of coronary arteries using basic ultrasound mode (B‐mode, or bright mode) will not pick up any flow motion. They do not interfere with each other. The flow velocity measurements were made at the same vessel site at baseline and during adenosine‐induced hyperemia (Fig. 1C). Measurements were averaged from three consecutive cardiac cycles.

Coronary blood flow was calculated using the following formula: Flow_CBF_ (mL/min) = ((π/4) × D^2^ × VTI × HR)/1000 (Katz et al. 2011) where D is the internal coronary diameter (in mm) measured in B‐mode ultrasound images, VTI is the velocity–time–integral (in mm), or area under the curve of the Doppler blood flow velocity tracing, and HR is heart rate. Coronary flow reserve (CFR) = CBF_adenosine_/CBF_baseline_ where CBF_adenosine_ is the peak coronary flow measured after 0.2 mg adenosine (in 500 *μ*L saline) femoral vein intravenous injection. The peak CBF was usually reached within 1½ mins after injection.

After peak CBF was reached, the effect of adenosine on cardiac function and coronary artery size were measured again.

### Statistical analysis

One‐way ANOVA and Bonferroni's multiple comparison post test was used to compare between mouse groups in all data set.

## Results

**Figure 2:**
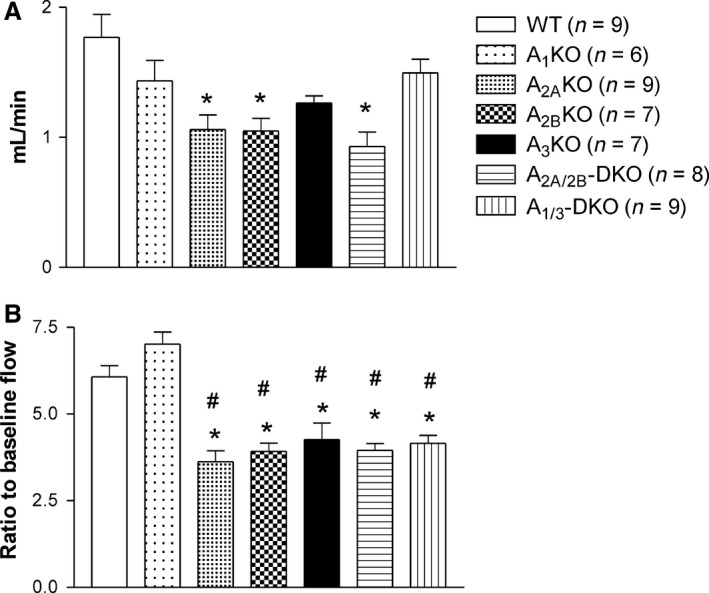
Coronary blood flow (A) and coronary reserve after bolus adenosine i.v. injection (B) for adenosine knockout mice and their wild type control. *Significantly different from WT; ^#^A_1_KO. *P* < 0.05.

**Figure 3:**
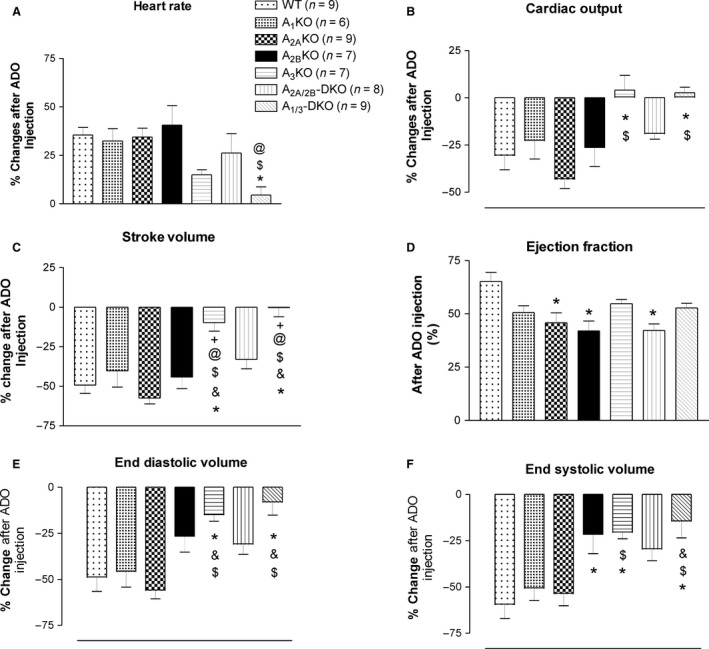
The effects of bolus adenosine i.v. injection on heart rate (A), cardiac output (B), stroke volume (C), ejection fraction (D), end diastolic volume (E), and end systolic volume (F) in adenosine knockout mice and their wild type control. *Significantly different from WT, ^&^A_1_KO, ^$^A_2_AKO, ^@^A_2_BKO, ^+^A_2_A/_2_B‐DKO. *P* < 0.05.

All baseline CBF and cardiac function parameters are presented in Table 1. CBF after adenosine injection and coronary reserve (CBF after adenosine injection/baseline CBF) are presented in Figure 2. For cardiac functions, data are presented as % changes (increase or decrease) after adenosine injection, except ejection fraction (EF), which was its original value after adenosine injection (Fig. 3). Comparisons were made between mouse groups.

**Table 1:** Baseline cardiac and coronary artery blood flow values from adenosine receptor knockout mice and their wild‐type control

Baseline value	Wild type (*n* = 9)	A_1_KO (*n* = 6)	A_2A_KO (*n* = 9)	A_2B_KO (*n* = 7)	A_3_KO (*n* = 7)	A_2A/2B_‐DKO (*n* = 8)	A_1/3‐_DKO (*n* = 9)
Coronary flow (mL/min)	0.31 ± 0.099	0.21 ± 0.07	0.30 ± 0.08	0.27 ± 0.05	0.31 ± 0.07	0.27 ± 0.08	0.37 ± 0.10 &
Heart rate (BPM)	452 ± 32	444 ± 29	484 ± 68	420 ± 46	442 ± 18	466 ± 89	498 ± 62
Stroke volume (*μ*L)	36.72 ± 7.61	24.93 ± 3.56^a^	31.13 ± 8.53	28.12 ± 5.11	33.52 ± 3.53	22.94 ± 4.71^a^	32.06 ± 7.88
Cardiac output (mL/min)	15.97 ± 3.13	10.98 ± 1.14^a^	14.66 ± 2.93	11.87 ± 1.65^a^	14.82 ± 1.63	10.56 ± 2.21^a^$%	15.70 ± 3.27 &+
Ejection fraction (%)	55.37 ± 7.49	46.01 ± 5.70	46.49 ± 8.34	54.57 ± 7.57	51.67 ± 4.2	43.15 ± 5.82^a^@	48.18 ± 6.74
EDV (*μ*L)	66.55 ± 12.02	57.48 ± 8.37	62.75 ± 11.56	49.98 ± 6.81	65.11 ± 7.11	53.15 ± 7.88	67.94 ± 20.67
ESV (*μ*L)	29.82 ± 8.12	31.91 ± 6.40	36.34 ± 11.78	24.39 ± 4.32	31.59 ± 5.51	30.21 ± 5.30	35.89 ± 14.33

a Significantly different from WT, ^&^A_1_KO, ^$^A_2A_KO, ^@^A_2B_KO, ^%^A3KO, ^+^A_2A2B_DKO; *P* < 0.05.

### Coronary flow

Baseline coronary flow was not different between all of the AR KOs and the wild‐type (WT) mice (Table 1), although A_1/3_ DKO mice showed higher baseline flow than A_1_ KO mice. Only the A_2A_ KO, A_2B_ KO, and A_2A/2B_ DKO mice demonstrated significant differences from WT in response to bolus adenosine injection (Fig. 2A), while coronary reserve in all single and DKO mice was significantly reduced except when A_1_ KO mice (Fig. 2B) were compared to WT.

### Heart rate

Baseline HR was not different between all groups of mice (Table 1). There were two phases of HR responses after bolus injection of adenosine. Initially, HR decreased instantly (for the first 20–30 sec) and significantly in all mice (from about 470 bpm to 150 bpm), except in A_1_ KO and A_1/3_ DKO mice. Complete atrioventricular (AV) block was also observed in most mice except A_1_ KO and A_1/3_ DKO mice. HR increased during the second phase. When compared between groups by their % changes in HR at the second phase, only A_1/3_ DKO mice demonstrated significantly less change from WT, A_2A_ KO, and A_2B_ KO mice (Fig. 3A).

### Stroke volume

Baseline stroke volume (SV) was significantly lower in A_1_ KO and A_2A/2B_ DKO mice (Table 1). Bolus adenosine injection induced a significant decrease in SV in WT mice. When compared between groups, only A_3_ KO and A_1/3_ DKO mice were significantly different from other groups (Fig. 3C).

### Cardiac output

Baseline cardiac output (CO) decreased significantly in A_1_ KO, A_2B_ KO, and A_2A/2B_ DKO mice (Table 1) when compared to WT mice. Bolus adenosine injection decreased CO. When compared between groups, the changes in CO in A_3_ KO and A_1/3_ DKO mice were significantly lower than WT and A_2A_ KO mice (Fig. 3B).

### Ejection fraction

Compared to WT mice, baseline EF decreased only in A_2A/2B_ DKO mice (Table 1). After bolus adenosine injection, EF increased in WT (from 55.37 ± 7.49% to 65.18 ± 12.79%), and the EF of A_2A_ KO, A_2B_ KO, and A_2A/2B_ DKO mice was lower than WT mice after adenosine injection (Fig. 3D).

### End‐diastolic volume

There was no difference in baseline end‐diastolic volume (EDV) between all mice (Table 1). After adenosine injection, EDV decreased. However, EDVs decreased less in A_3_ KO and A_1/3_ DKO mice after adenosine injection when compared to other mouse groups. (Fig. 3E).

### End‐systolic volume

Similar to EDV, there is no difference in baseline end‐systolic volume (ESV) between all mice (Table 1), and ESV decreased after adenosine injection (Fig. 3F). When compared between mouse groups, the decrease in ESV was less in A_2B_ KO, A_3_ KO, and A_1/3_ DKO mice when compared to WT mice. The decrease in ESV in A_3_ KO and A_1/3_ DKO mice was also significantly less than in A_1_ KO mice, and the decrease in A_1/3_ DKO mice was less when compared to A_2A_ KO mice (Fig. 3F).

## Discussion

This is the first in vivo study looking into both the cardiac and coronary flow responses of adenosine in all existing AR KO mice (single and double). Although this has not been done in all four AR KOs, the authors have done isolated mouse coronary artery study in A_2A_ KO mice (Teng et al. 2008). In addition, several studies from our lab and others using isolated mouse hearts have clearly demonstrated AR‐mediated coronary vasodilation in mice (Morrison et al. 2002; Teng et al. 2008; Headrick and Lasley 2009; Sanjani et al. 2011). The technique we used here is not new, though rarely used in measuring mouse coronary artery flow due to technical difficulty. Furthermore, previous studies mostly focused on one or two strains of adenosine receptor KO mice. In this study, using all four adenosine KO mice give us a unique prospective of the cardiac and coronary effects of adenosine in live animals, which has never been done in the past. The baseline coronary flow was well maintained in all KO mice in this study; however, their responses to adenosine bolus injection were not the same. As expected, the coronary flow responses to adenosine were lower in A_2A_ KO, A_2B_ KO, and A_2A/2B_ DKO mice compared to WT mice (Fig. 2A), which supports our and others ex vivo model findings that both A_2A_ and A_2B_ ARs play major roles in adenosine‐induced coronary vasodilation. Interestingly, except for A_1_ KO mice, all other KO mice showed decreases in coronary reserve (Fig. 2B). The major determinant for CBF is oxygen demand of the heart; therefore, the effect of adenosine on HR, contractility, and wall tension can all possibly be affecting CBF and reserve. A_1/3_ DKO mice demonstrated relatively higher baseline CBF (Table 1) when compared to other groups (except A_1_ KO mice), indicating possibly higher baseline cardiac work. However, ventricular pressure was not measured in our experiments (hence no index of cardiac work); therefore, it is difficult to interpret these results and the physiological significance of these findings. Nevertheless, the importance of A_2A_ and A_2B_ ARs in CBF regulation was confirmed in vivo in mice.

In addition to coronary circulation, all four ARs were also found in cardiac tissue (Lasley and Smart 2001; Morrison et al. 2006). In isolated heart tissue, adenosine produces a negative inotropic effect in atrial and ventricular myocardium (Dobson 1983; Belardinelli et al. 1989). The A_1_ AR was found to exert a negative inotropic effect by inhibiting *β*‐adrenergic effects, while the A_2A_ AR modulated the *β*‐adrenergic inhibiting effect of the A_1_ AR (Tikh et al. 2006; Chandrasekera et al. 2010). The A_2B_ AR demonstrated moderate direct, positive inotropic effects (Chandrasekera et al. 2010). Therefore, it is not surprising to observe lower baseline SV and CO in A_2A/2B_ DKO mice (Table 1). Future studies on the cross‐talk between ARs and *β*‐adrenergic receptors are warranted. However, it is surprising that the baseline SV and CO also decreased in A_1_ KO mice. Previous studies in isolated heart experiments in A_1_ KO mice did not show baseline changes in contractility when compared to WT mice (Morrison et al. 2000, 2004; Tawfik et al. 2006). The reason for this discrepancy is not clear. However, we speculate that this decrease in baseline SV and CO in A_1_ KO mice may be due to compensatory changes in another vascular system(s) that affects their hemodynamic regulation. For instance, upregulation of the A_2B_ AR was reported from our lab in the mesenteric arteries of A_1_ KO mice (Teng et al. 2013), but their influence on regional blood flow may affect systemic circulation, and this has not been studied. Furthermore, our previous ex vivo studies on coronary flow regulation in A_2A_ and A_2B_ KO mice demonstrated up‐regulation of either AR, if the other was genetically deleted (Teng et al. 2008; Sanjani et al. 2011). Therefore, compensatory responses in these KO mice cannot be ignored. Although the A_3_ AR has been proven to protect cardiac function in ischemia‐reperfusion injury (Cerniway et al. 2001; Harrison et al. 2002), it did not seem to directly affect cardiac contractility in isolated heart preparations (Harrison et al. 2002; El‐Awady et al. 2013) that measured left ventricular function. However, the A_3_ AR induces positive inotropy in atrial tissue through regulating the ryanodine receptor and calcium/calmodulin‐dependent kinase II (CaMKII) (Yuan et al. 2008). Furthermore, A_3_ AR overexpressing mice also demonstrated an increase in left atrium size, suggesting increased ESV (Fabritz et al. 2004). Based upon the Frank–Starling mechanism, an increase in right atrium contraction increases preload (i.e., end diastolic volume) and subsequently increases SV. This is supported by our study where A_3_ KO and A_1/3_ DKO mice were the only groups with reduced EDV and ESV changes (Fig. 3E and F) that were reflected in the reduction in SV and CO (Fig. 3B and C).

A significant decrease in HR after application of adenosine was observed in ex vivo animal models in our lab and others and is well documented as an A_1_ AR‐mediated effect, including both direct (shortening action potential) and indirect (inhibiting *β*‐adrenergic effect) (Headrick et al. 2000; Lerman et al. 2001; Dhalla et al. 2003; Kirchhof et al. 2003; Mustafa et al. 2009) effects. In healthy subjects, bolus injection of adenosine briefly reduces heart rate and induces AV block, followed by a more sustained increase in HR (Sylven et al. 1989), while infusion of adenosine induces a dose‐dependent increase in HR (Biaggioni et al. 1986) that may be due to an adenosine‐induced decrease in blood pressure (reflex tachycardia). A previous in vivo study in anesthetized mice also demonstrated a similar biphasic HR response after bolus adenosine injection (Koeppen et al. 2009). Because of the significant adenosine‐induced decrease in blood pressure found in this study, it is reasonable to presume that the secondary increase in HR (about 30 sec after injection) after the initial decrease is due to reflex tachycardia. In our study, we observed the same biphasic changes in HR after bolus adenosine injection in all mouse strains except with A_1_ AR deletion (A_1_ KO and A_1/3_ DKO), which only shows that HR increases (the second phase) about 30 sec after the injection. Surprisingly, A_3_ AR deletion (A_3_ KO and A_1/3_ DKO mice) did not show the second phase of HR increase (Fig. 3A). This is the first report of such a finding in A_3_ KO mice. A_3_ AR overexpressing mice showed profound resting bradycardia, AV block, and incessant bradycardia–tachycardia syndrome, but return to normal rhythm during exercise (Fabritz et al. 2004). Unlike the A_1_ AR, its effect was not *β*‐adrenergic mediated and is possibly mediated through Rho kinase (Fabritz et al. 2004). A previous in vivo study of intracerebral‐ventricular injection of adenosine analogs suggested that there is a population of A_3_ ARs present in the CNS whose activation induces a decrease in blood pressure with no change in HR (Stella et al. 1998). Since it is still unclear how the central and peripheral mechanisms of adenosine receptor‐dependent regulation of blood pressure and HR act in concert, further studies are needed to clarify the role of ARs in HR regulation.

Another interesting finding in this study is the influence of the A_2A_ and A_2B_ ARs on EF. In a mathematic model, EF equals to SV divided by EDV and is an important indicator for cardiac function. In all the KO mice we studied, A_2A/2B_ DKO mice were the only KO model that showed significantly lower EF than WT control (Table 1). Despite a reduction in SV and CO after adenosine injection, EF increased to 65.19 ± 12.79% (Fig. 3D) in WT mice from a baseline of 55.37 ± 7.49% (Table 1). After adenosine injection, only A_2A_ KO, A_2B_ KO, and A_2A/2B_ DKO mice demonstrated significant differences in EF (45.85 ± 13.90, 41.98 ± 12.40, and 42.21 ± 8.65%, respectively) when compared to WT (Fig. 3D). In isolated heart studies, both the A_2A_ and A_2B_ AR were found to increase contractility (Dobson 1983; Tikh et al. 2006; Chandrasekera et al. 2010). However, increases in HR, like we observed in this study after adenosine injection, has been shown to decrease SV, EDV, and ESV due to decreases in ventricle refilling time (Erbel et al. 1984). Combined effects of increased inotropy and reduced ventricle refilling time (increase in HR) may create stiffness in the ventricles (i.e., increasing end‐diastolic pressure without increasing EDV). Furthermore, A_2A_ AR stimulation has been shown to decrease venous resistance (Nekooeian and Tabrizchi 1996, 1998), possibly reducing venous return and reducing EDV further, increasing EF. However, the A_3_ AR (and possible the A_2B_ AR) seems to play a prominent role in adenosine‐induced EDV and ESV reduction by constricting the left atrium as mentioned above (Fig. 3E and F). It becomes very difficult to interpret the in vivo data in relation to possible mechanism(s) of A_2A_ and A_2B_ AR‐mediated improvement in EF. Further studies are needed to address this paradox using isolated tissues with molecular approach.

In conclusion, using KO mice, we confirmed that both the A_2A_ and A_2B_ ARs are involved in adenosine‐induced increases in CBF. Bolus injection of adenosine at a dosage of 0.2 mg/mouse (about 10 mg/kg) decreased CO and SV and may be A_2B_ and/or A_3_ AR‐mediated; however, the A_1_ and A_2A_ ARs also play roles in overall cardiac function, such as in HR and EF regulation. These data further provide evidence that the combination of AR KO mice and the use of the Doppler/ultrasound technique to measure cardiac and coronary function in live animals is a powerful translational tool in studying the cardiovascular effects of adenosine, especially in disease states, in the future.

### Limitations of the study

The resolution of *Vevo* system is 30 *μ*m while the size of the mouse left coronary artery is about 200 *μ*m measured by the system. The standard errors of coronary artery were quite large in some cases. Therefore, it is hard to definitively state small changes. To simplify, some previous studies only used VTI or max flow as an indicator of coronary flow to calculate the reserve (Wikstrom et al. 2005, 2008), which is not suitable in our study that requires actual flow measurement to compare baseline blood flow. Nevertheless, in general, the baseline coronary artery size is about 200 and 220–230 *μ*m after adenosine injection in KO mice. The difference is smaller than the resolution stated. Therefore, we cannot state with certainty the adenosine effect on size of the coronary artery.

Although all AR KO mice we studied here have been in existence for some time, not all of them have their cardiovascular phenotype evaluated. In few reports, only A_2A_ KO mice have been shown to be hypertensive with some neurological disorder (Ledent et al. 1997). To the best of our knowledge, there is no report of other AR KO mice demonstrating baseline blood pressure deviation from WT. Since blood pressure affects cardiac work and subsequent coronary blood flow, the lack of information on blood pressure in our study (our current ultrasound equipment setting does not allow us to measure blood pressure simultaneously) did limit our full interpretation of the cardiovascular function (s) of adenosine. Nevertheless, the utilization of all AR KOs still provides valuable validation of previous ex vivo study and some insight into possible future pharmacological studies in relation to the cardiovascular effects of adenosine with potential for translational studies.

## Conflict of Interest

None declared.
